# Mining and analysis of multiple association rules between the Xining loess collapsibility and physical parameters

**DOI:** 10.1038/s41598-020-78702-7

**Published:** 2021-01-12

**Authors:** Zhikun Li, Xiaojun Li, Yanyan Zhu, Shi Dong, Chenzhi Hu, Jixin Fan

**Affiliations:** 1 Xi’an University of Science and Technology, Xi’an, 710054 Shaanxi China; 2 Key Laboratory for Geo-Hazards in Loess Area, MLR, Xi’an, 710054 Shaanxi China; 3 Hebei Expressway Zhangcheng Chengde Management Office, Chengde, 067000 Hebei China; 4 Engineering Research Center of Highway Infrastructure Digitalization of Ministry of Education, Chang’an University, Xian, 710064 Shaanxi China; 5 Qinghai Bureau of Coal Geology, Xining, 810029 Qinghai China

**Keywords:** Engineering, Civil engineering

## Abstract

Collapsibility determination in loess area is expensive, and it also requires a large amount of experimentation. This paper aims to find the association rules between physical parameters and collapsibility of the loess in Xining through the method of data mining, so to help researchers predict the collapsibility of loess. Related physical parameters of loess collapsibility, collected from 1039 samples, involve 13 potential influence factors. According to Grey Relational Analysis, the key influence factors that lead to collapsing are identified from these potential influence factors. Subsequently, take the key influence factors, δs (coefficient of collapsibility) and δzs (coefficient of collapsibility under overburden pressure) as input items, and use the Apriori algorithm to find multiple association rules between them. Then, through analysing the results of association rules between these key influence factors and collapsibility, the evaluation criteria for collapsibility in this area is proposed, which can be used to simplify the workload of determining collapsibility. Finally, based on these research results, recommendations for projects construction were made to ensure the safety of construction in the area.

## Introduction

In the loess area, deformation caused by collapsibility is a major distress mode in engineering construction. With the loess immersion under pressure, the structure is destroyed, and the pores gradually narrow, eventually leading to loess collapsible. Physical parameters have an important influence on the collapsibility of loess soils. The water indicators, density, pore, burial depth, geostatic stress, and physical characteristics can all influence collapsibility of loess soils^1^. It is difficult for designers to determine which factor to use to characterize collapsibility, and what kind of standards should be applied to accurate identification of loess stability.

Until recently, the collapsible mechanism was still the focus of loess research. Due to the special structure and characteristics of loess, the cause of collapsibility is inconclusive. In the early research, the researchers believed that the factors affecting collapsibility were single, and theories including Soluble salt hypothesis and Colloidal deficiency hypothesis were proposed. As research continues, researchers have discovered that there are many complex factors leading to collapsibility^2–4^. By studying the influence of soil characteristics on collapsibility, it was found that the possibility of collapse is related to the pore ratio, water content, density and other indicators of the undisturbed soil. These conclusions have also been confirmed in subsequent studies^5–9^. For example, as the initial water content and dry density increase, collapsibility tends to decrease^7–11^. It is widely accepted that the difference in collapsing is also reflected in the microstructure of the soil^12,13^. Numerous studies have shown that changes in the accumulation state of loess can also affect collapsibility^14^. Based on these results, the researchers tried to use some parameters of the soil to predict collapsibility. In regional studies, the predicted results are effective in general which in conventional geotechnical engineering practices^15^.

Collapsibility is still one of the most difficult engineering geological problems to predict. According to incomplete statistics, in Northwest China, there are more than 400 large-scale ground subsidences caused by the collapse of loess. In the Heifangtai area of Gansu, China, due to the deformation of the foundation, each family has renovated their houses twice on average^16^. Therefore, how to correctly evaluate the collapsibility level of loess has great engineering significance. Currently, there are two types of evaluation coefficient that identify the collapsibility of loess: δzs and δs. Both are usually obtained from the indoor immersion compression test. Compared with the field immersion deformation test, although the associated with far less expense, the huge amount of experiments and the complicated process are still the difficulties in predicting collapsibility at this stage^2^. In recent years, researchers have tried to use machine learning or artificial intelligent methods to predict collapsibility^17,18^. But before this, there are few studies on the large and multiple association rules between influencing factors and collapsibility.

Xining, Qinghai province of China is located on the edge of the Qinghai-Tibet Plateau. It is an important city in the “ The Belt and Road ” policy. In the past project construction in this area, a large amount of data has been accumulated. How to summarize these data to serve the future project construction has become a common concern for many engineers and researchers. In Xining, Qinghai province, rarely the method of big data analysis has been used on the studies of the engineering characteristics of loess. Therefore, this paper uses the Apriori algorithm to mine the relationship between physical parameters and collapsibility levels in 1039 loess samples, aiming to discover multiple association rules between them. In the analysis of these association rules, we can access much quantitative information, among which the evaluation criteria for collapsibility in this area are required. It can be used to simplify the workload of determining collapsibility. To achieve that, three steps are required: (1) identify the representative indicators that lead to collapsibility from 13 potential factors; (2) analyze the association rules of each factor with δs(coefficient of collapsibility) and δzs(coefficient of collapsibility under overburden pressure) separately; and (3) providing the evaluation criteria for collapsibility and constructive recommendations for projects construction based on the results obtained.

The following sections describe this procedure and methodologies in detail. The first step is to collate the data in the engineering survey report, thereby establishing a dataset that includes 13 potential factors and δs and δzs. Then, the original data has to be preprocessed, including reducing noise and normalization, discretization.The third step is to identify the key factors that lead to collapsibility, which use the method is Gray relational analysis. Subsequently, take key factors, δs and δzs as input item, and the Apriori algorithm is used to find multiple association rules. Compared with previous research, the results obtained from information mining based on big data are more reliable. Finally, based on the analysis results of the association rules, the evaluation criteria for collapsibility can be proposed, which can provide assistance to the engineering geological survey in the area, thereby simplifying the workload of indoor experiments.

## Study site and data

### Description of the study site

The topography of Xining, Qinghai province of China is located in the transitional zone between the Loess Plateau Plateau and the Qinghai-Tibet. During the Cenozoic, the area accumulated thick and continuous loess, nearly 25 m^19^. The study area is located in the Chengbei District, Xining, covering an area of about 137.7 km^2^ between longitudes 36°64′42″–36°69′65″N and latitudes 101°74′29″–101°76′45″E. The altitude increases from Northwest to Southeast and varies in the range from 2755 to 2215 m. The climate of the study area is characterized by the Alpine plateau climate; low pressure, low rainfall, large evaporation, long freezing period, large temperature difference between day and night. According to the China meteorological administration, the temperature in the region varies between − 26.6° C in winter and 38.7° C in summer, and the annual average is 5.7° C. The rainfall is about 7 mm in winter and 255 mm in summer, and the annual average is 371 mm. According to the engineering geological survey report, Qauternary strata is the major strata in the region, and all samples are Q4 loess. The soil characteristics are: silty soil with collapsibility, high compressibility and low strength; the pores are arranged in disorder, and there is calcium powder on the hole-wall^20,21^.

### Data

**Figure 1:**
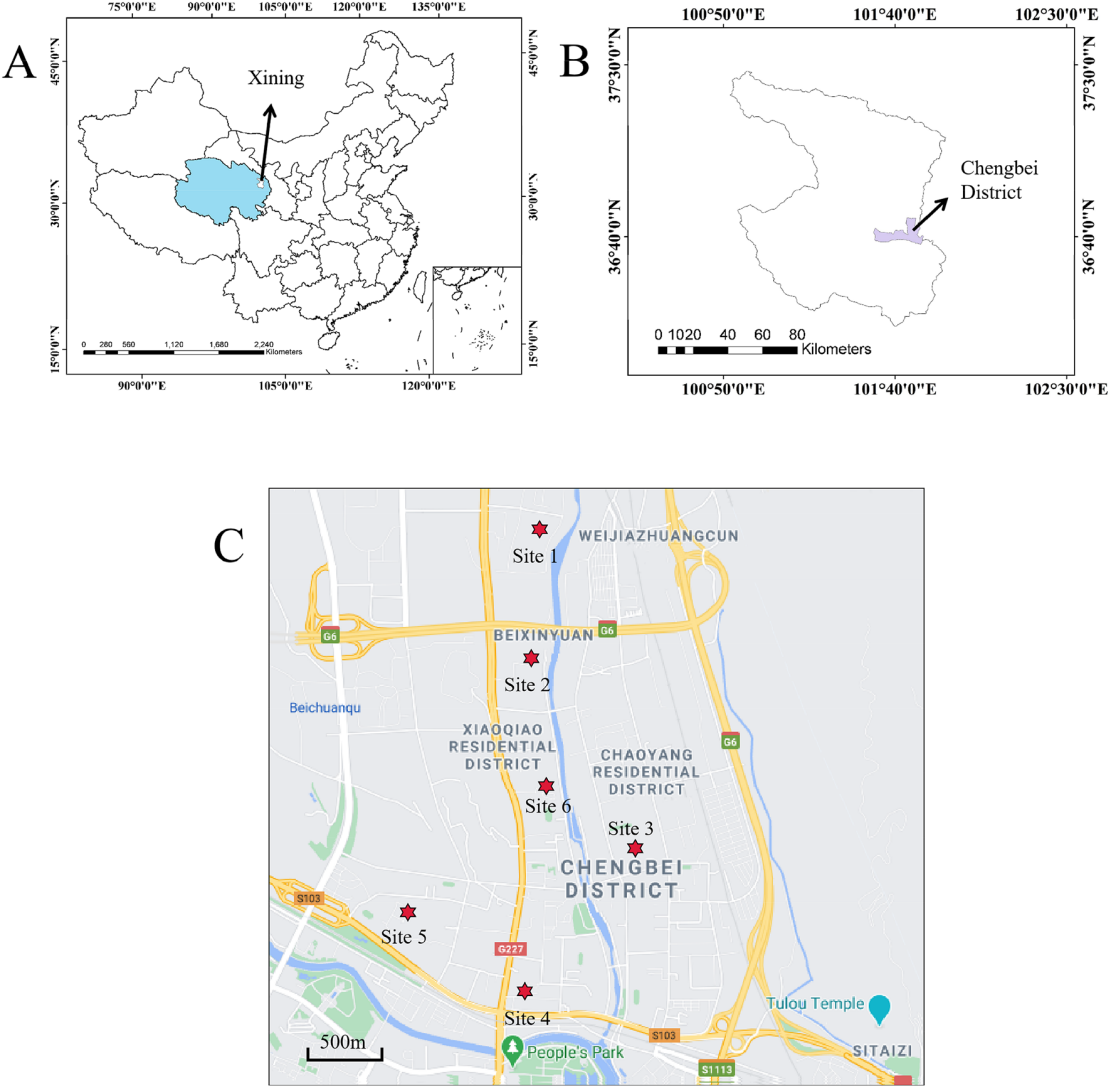
Geographic location of the data collection. Created by Arcgis10.6 (https://www.arcgis.com/index.html) and Baidu map15.0. (https://map.baidu.com).

In this study, the data originated from six construction projects, which were used to mine the association rules of collapsibility of loess. According to the Raida criterion, all data meet the statistical requirements. The specific location of those projects is shown in Fig. 1 and Table 1.

**Table 1:** Detailed data on study sites.

Site	Data sources	Elevation(m)	Mean groundwater level(m)
1	Stone Leixincun	2230	3.4
2	No.4 Middle School	2258	4.6
3	Minhui City	2245	6.5
4	Guotai Wangzuo	2237	2.8
5	No.5 Automobile Factory	2267	3.9
6	Shenna Middle School	2244	4.6

**Figure 2:**
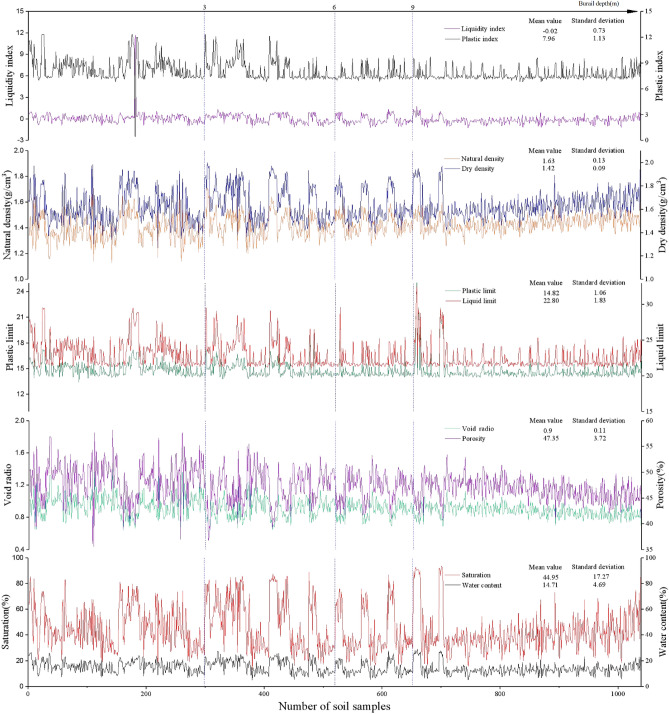
Physical parameters of soil samples.

There are many physical parameters that lead to collapsibility. These potential factors can be divided into six categories: water indicators, density, pore, burial depth, geostatic stress, and physical characteristics. In this study, the dataset included 13 potential influence factors for 1039 samples (The details are shown in Fig. 2), which originated from six construction projects of the Chengbei district. Subsequently, those original data need to be preprocessed, including reducing noise and normalization, discretization.

### Collapsibility of loess soils

**Figure 3:**
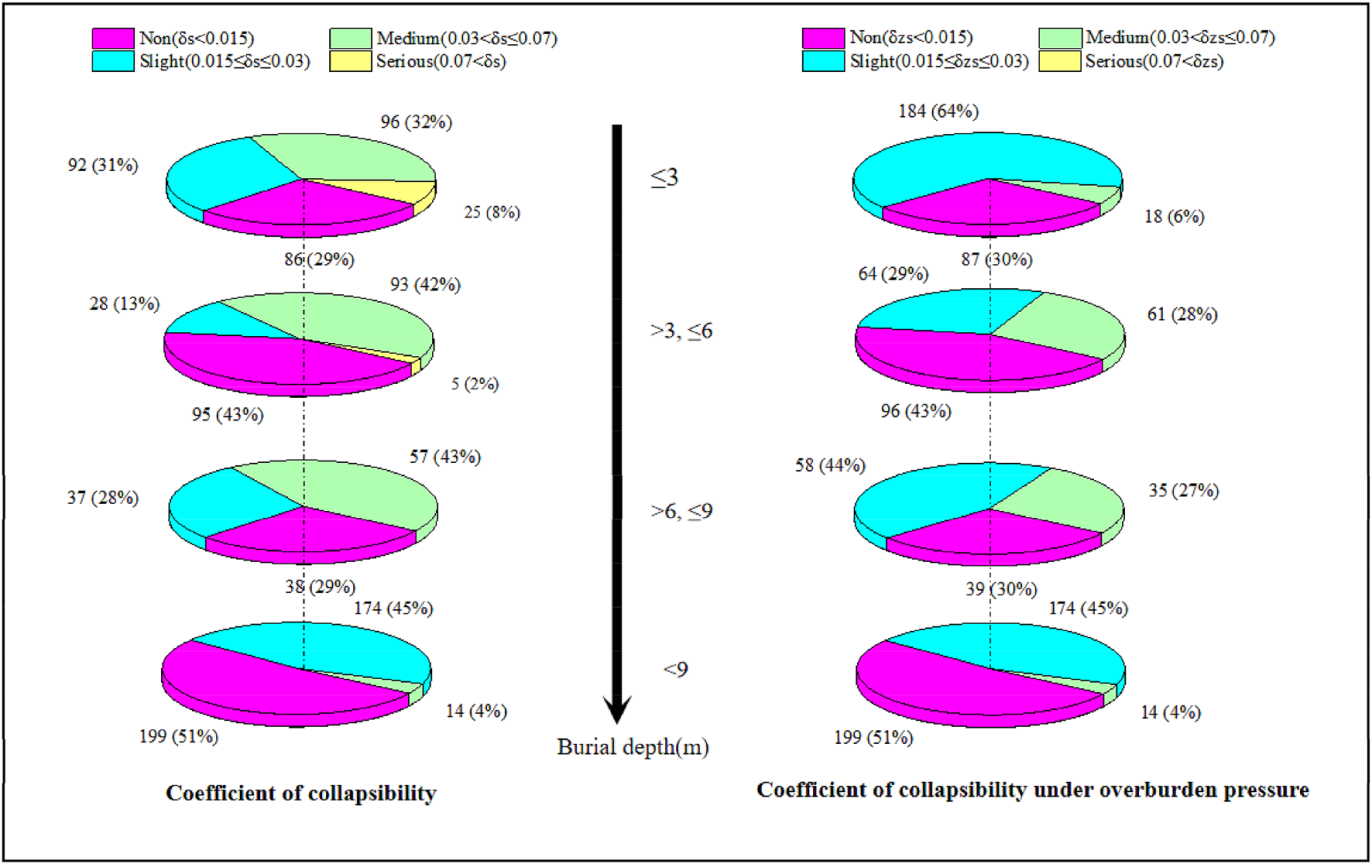
Collapsibility level (δs and δzs) distribution under different burial depths.

Loess has been used as a foundation in various construction projects for a long time. The collapsibility of loess has always been a typical engineering geological problem in the loess region. The collapsibility of loess often causes huge damage to the engineering construction activities in its distribution area and is extremely destructive to engineering buildings. δs and δzs are important indicators for evaluating the collapsibility of loess. Both of them play important roles in engineering construction in the loess area. Details of δs and δzs in the study area can be seen in Fig. 3.

#### Coefficient of collapsibility

An index for measuring the degree of collapsibility of a soil mass after immersion in water under a given pressure. According to the test of indoor confined compression. The definition of the coefficient of collapsibility(δs) can be represented as:

1 $$\updelta s = \frac{{h_{p} - h_{p}^{\prime } }}{h0} $$

where *h*_*0*_ is the soil thickness to maintain natural humidity and structure. *h*_*p*_ is the soil thickness after subsidence and stabilization when the soil sample is pressurized to *p* (mm); *h*_*p*_^*′*^ is the thickness (mm) of the soil sample after being stabilized under pressure and sinking and stable under the action of water immersion. The pressure p is determined from the bottom of the foundation (preliminary survey from 1.5 m below the ground) 200 kPa within 10 m, and the saturated self-weight pressure of the overlying soil under 10 m to the top of the non-collapsible soil layer (300 kPa is still used when it is greater than 300 kPa).

#### Coefficient of collapsibility under overburden pressure

The ratio of the subsidence of the loess sample to the original height of the sample under the action of saturated self-gravity of the soil. It is an important index for judging self-weight collapse. Coefficient of collapsibility under overburden pressure (δs) can be presented as follows:

2 $$\updelta sz = \frac{{hz - h_{z}^{\prime } }}{{h_{0} }} $$

where *h*_*z*_ is the thickness (cm) when the soil sample is pressurized to the saturated dead weight pressure corresponding to the overlying soil and subsidence is stable. *h*_*z*_^*′*^ is the thickness (cm) of the soil sample after pressure stabilization, under the action of immersion in water, and after sinking and stabilization.

## Research methodology

### Grey Relational analysis

Grey relational analysis is a method that uses Grey Relation Order (GRO) to describe the strength of the association, which was proposed by Tan and Deng. This method is widely used in industry, economics, management and other disciplines, and has achieved remarkable results. In this paper, the GRA algorithm is used to find out the significant factors in each category, and then these factors are the input items of the Apriori algorithm.

### Apriori algorithm

**Figure 4:**
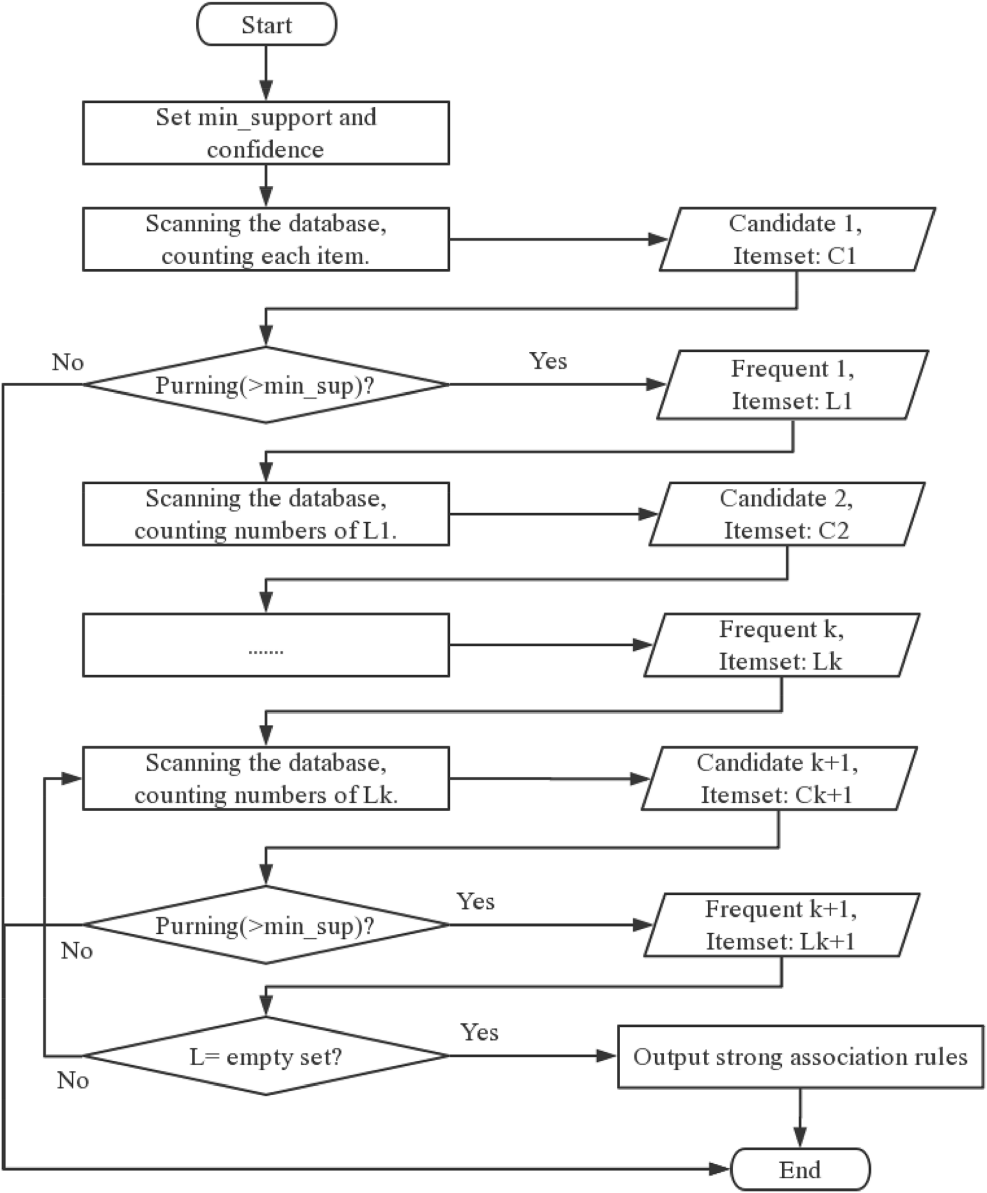
Flowchart of apriori computer procedure.

Association rule analysis is necessary for data mining. By using association analysis to find frequent itemsets in the data, the structural characteristics of the data are revealed. Apriori algorithm is a classic algorithm for finding frequent itemsets and generating association rules based on this. Its essence is an iterative method of layer-by-layer search, and each search is divided into two stages: generating candidate sets and checking support. In the application of the Apriori algorithm, researchers can adjust the thresholds of the screening indicators, including Support and Confidence, thereby ensuring the practicability of the results. Hence, in this paper, the Apriori algorithm was used to investigate the correlation between influencing factors and collapsible levels. The implementation steps of the Apriori algorithm are shown in Fig. 4.

## Results and discussion

### Determine the model input item

The 13 potential factors can be divided into six categories according to characteristics, including the water indicators, density, pore, burial depth, geostatic stress, and physical characteristics. Due to its large amount of data, it is necessary to use correlation analysis to identify the most important factors in each category.

**Figure 5:**
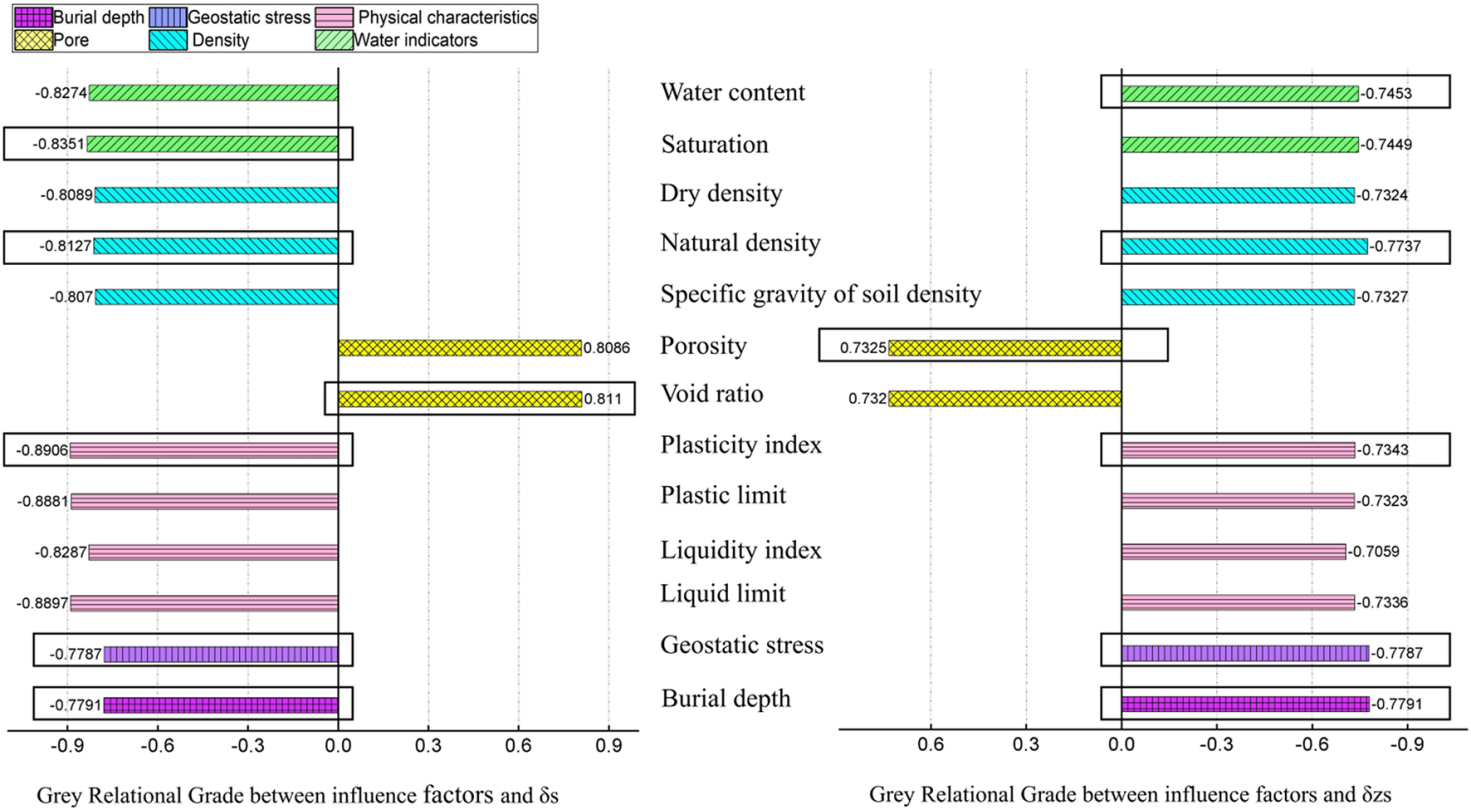
Grey relational grades between influence factors and δs or δzs.

The gray correlation level of each influencing factor and δs or δzs is shown in Fig. 5. As for pore, the porosity and void ratio had positive correlations with collapsibility, which may be interpreted as the pores increasingly in the loess soils allowing collapsibility to seriously. Physical characteristics had negative correlations with the collapsibility. As can be seen from the results of the Grey Relational Grades, I_P_ is the most important significant influence factor within physical characteristics group. As for density, the natural density had the maximum GRG. In water indicators, the saturation has a higher correlation than water content for coefficient of collapsibility(δs), while the reverse was true for coefficient of collapsibility under overburden pressure(δzs).

Based on the results of Grey Relational Grades, select the most important factors in each category as input item of the Apriori algorithm: (1) for δs: saturation, natural density, void ratio, plasticity index, geostatic stress, burial depth. (2) for δzs: water content, natural density, porosity (for statistical purposes, convert this to void ratio), plasticity index, geostatic stress, burial depth. Then, the Apriori algorithm is used to mine association rules.

### Result analysis of Apriori algorithm

In order to be used as an input item of the Apriori algorithm, the data should be preprocessed. The first step is normalization, uses the Min–max normalization method. The second step, discretization, uses a clustering algorithm to separate each factor into four categories according to different ranges. The preprocessing results are shown in Table 2. It can be seen that each factor is divided into four quantization types. For example, D1 means the Level1 (≤ 1.54 g/cm^3^) of the natural density.

**Table 2:** Classification standards for collapsibility and key influence factors.

Category	Key Influence factor	Description of each level	Threshold value	Normalized threshold value	Sample size	Factor code
Water indicators	Saturation(%)(δs)	Super high saturation	> 67.6	(0.678, 1]	133	S4
		High saturation	> 48, ≤ 67.6	(0.435, 0.678]	237	S3
		Medium saturation	> 33.5, ≤ 48	(0.254, 0.435]	378	S2
		Low saturation	> 0, ≤ 33.5	(0, 0.254]	291	S1
	Water content(%)(δzs)	Super high water content	> 20.4	(0.644, 1]	136	W4
		High water content	> 15.3, ≤ 20.4	(0.434, 0.644]	273	W3
		Medium water content	> 11, ≤ 15.3	(0.256, 0.434]	403	W2
		Low water content	> 0, ≤ 11	(0, 0.256]	227	W1
Density	Natural density(g/cm³)	Super high density	> 1.8	(0.726, 1]	132	D4
		High density	> 1.66,≤ 1.8	(0.534, 0.726]	218	D3
		Medium density	> 1.54, ≤ 1.66	(0.369, 0.534]	375	D2
		Low density	> 0, ≤ 1.54	(0, 0.369]	314	D1
Pore	Void ratio(δs)	Super high void ratio	> 1.059	(0.602, 1]	90	E4
		High void ratio	> 0.929, ≤ 1.059	(0.448, 0.602]	324	E3
		Medium void ratio	> 0.816, ≤ 0.929	(0.314, 0.448]	413	E2
		Low void ratio	> 0, ≤ 0.816	(0, 0.314]	212	E1
	Void ratio(δzs)	Super high void ratio	> 1.012	(0.546, 1]	168	EO4
		High void ratio	> 0.890, ≤ 1.012	(0.401, 0.546]	387	EO3
		Medium void ratio	> 0.789, ≤ 0.890	(0.281, 0.401]	332	EO2
		Low void ratio	> 0, ≤ 0.789	(0, 0.281]	152	EO1
Depth	Burial depth(m)	More than 9m	> 9	(0.595, 1]	387	H4
		6 to 9m	> 6, ≤ 9	(0.277, 0.595]	132	H3
		3 to 6m	> 3, ≤ 6	(0.118, 0.277]	221	H2
		≤ 3m	> 0, ≤ 3	(0, 0.118]	299	H1
Geostatic stress	Geostatic stress(kpa)	Super high geostatic stress	> 252.3	(0.606, 1]	158	G4
		High geostatic stress	> 164.3, ≤ 252.3	(0.378, 0.606]	192	G3
		Medium geostatic stress	> 88.64, ≤ 164.3	(0.182, 0.378]	206	G2
		Low geostatic stress	> 0, ≤ 88.64	(0, 0.182]	483	G1
Physical characteristics	Plasticity index	Super plasticity	> 10.2	(0.823, 1]	46	P4
		High plasticity	> 8.2, ≤ 10.2	(0.645, 0.823]	286	P3
		Medium plasticity	> 7.3, ≤ 8.2	(0.578, 0.645]	311	P2
		Low plasticity	> 0, ≤ 7.3	(0, 0.578]	396	P1
Collapsibility	Coefficient of collapsibility	Serious collapsibility	> 0.070	–	30	C4
		Medium collapsibility	> 0.030, ≤ 0.070	–	260	C3
		Slight collapsibility	≥ 0.015, ≤ 0.030	–	331	C2
		Non-collapsibility	< 0.015	–	418	C1
	Coefficient of collapsibility under overburden pressure	Serious collapsibility	> 0.070	–		CO4
		Medium collapsibility	> 0.030, ≤ 0.070	–	138	CO3
		Slight collapsibility	≥ 0.015, ≤ 0.030	–	480	CO2
		Non-collapsibility	< 0.015	–	421	CO1

There are two kinds of association rules used for analysis in this paper, which is obtained by Apriori algorithm: (1) when circumstances are high confidence level, find the rules which have the highest support level. (2) the rule where the confidence is 100%.

For mining category (1), the results are shown in Table 3. It is worth pointing out that the thresholds of confidence and support here are 4% and 70%, respectively.

**Table 3:** Association rules between influence factors and collapsibility (high Support with relatively high confidence.

No.	If (influence factors)	Then (δs)	Support (%)	Confidence (%)
1	Void ratio = E1(≤ 0.816)	δs = C1 (non)	8.71	85.38
2	Density = D3(1.66–1.8 g/cm³)	δs = C1 (non)	7.70	73.39
3	Geostatic stress = G4(> 252.3kpa)	δs = C1 (non)	7.36	96.84
4	Geostatic stress = G3(164.3–252.3kpa)	δs = C2 (slight)	6.83	73.96
5	Saturation = S4(> 67.6%)	δs = C1 (non)	5.87	91.73
6	Density = D4(> 1.8 g/cm³)	δs = C1 (non)	5.63	88.64
7	Density = D4(> 1.8 g/cm³) and Saturation = S4(> 67.6%)	δs = C1 (non)	4.91	92.73
8	Density = D4(> 1.8 g/cm³) and Void ratio = E1(≤ 0.816)	δs = C1 (non)	4.86	87.07
9	Density = D2(1.54–1.66 g/cm³) and Geostatic stress = G3(164.3–252.3kpa)	δs = C2 (slight)	4.43	85.98
10	Void ratio = E1(≤ 0.816) and Saturation = S4(> 67.6%)	δs = C1 (non)	4.18	91.58
11	Void ratio = E2(0.816–0.929) and Geostatic stress = G4(> 252.3kpa)	δs = C1 (non)	4.18	95.60
12	Geostatic stress = G3(164.3–252.3kpa) and Saturation = S2(33.5–48%)	δs = C2 (slight)	4.18	82.65

No.	If (influence factors)	Then (δzs)	Support	Confidence
1	Density = D3(1.66–1.8 g/cm³)	δzs = CO1 (non)	7.65	72.94
2	Geostatic stress = G4(> 252.3kpa)	δzs = CO1 (non)	7.36	96.84
3	Geostatic stress = G3(164.3–252.3kpa)	δzs = CO2 (slight)	6.83	73.96
4	Void ratio = E01(≤ 0.789)	δzs = CO1 (non)	6.49	88.82
5	Density = D4(> 1.8 g/cm³)	δzs = CO1 (non)	5.72	90.15
6	Water content = W4(> 20.4%)	δzs = CO1 (non)	5.19	79.41
7	Density = D3(1.66–1.8 g/cm³) and Void ratio = EO2(0.789–0.890)	δzs = CO1 (non)	5.10	71.41
8	Density = D2(1.54–1.66 g/cm³) and Geostatic stress = G3(164.3–252.3kpa)	δzs = CO2 (slight)	4.43	85.98
9	Burial Depth = H1(< 3 m) and Plasticity index = P3(8.2–10.2)	δzs = CO2 (slight)	4.42	70.23
10	Density = D4(> 1.8 g/cm³) and Void ratio = EO1(≤ 0.789)	δzs = CO1 (non)	4.28	89
11	Geostatic stress = G4(> 252.3kpa) and Water content = W2(11–15.3%)	δzs = CO1 (non)	4.28	97.80
12	Void ratio = EO2(0.789–0.890) and Geostatic stress = G4(> 252.3kpa)	δzs = CO1 (non)	4.09	96.59

As mentioned above, the Support represents the probability that A and B occur simultaneously, and the meaning for the Confidence is the probability that B will occur if A occurs. By analyzing them, the specific influence of factor changes on the collapsibility can be obtained, so as to explore the fundamental principles of loess collapsibility. (1): For δs, Support (E1⇒C1) = 8.71%, which signifies that there are 8.71% of instances where void ratio ≤ 0.8106 and non-collapsibility appear at the same time. When Confidence (E1⇒ C1) is 85.38%, this indicates that if the void ratio is under 0.8106, the probability of non-collapsibility equals 85.38%. The Confidence (D4 ∩ S4⇒ C1) = 92.73% indicates that saturated soil with high natural density can reduce the probability of collapsible level. The confidence of (D2 ∩ G3⇒ C2) = 85.98% reveals that if the medium-density loess soil samples with geostatic stress at 164.3–252.3 kPa, the probability of slight collapsibility is only 4.43%. Comparing the Confidence (D4⇒ C1) = 88.64% and Confidence (D3⇒ C1) = 73.39%, it can be found that heavier natural density can decrease the risk of collapsibility from 15.34 to 0%. (2): For δzs, the Confidence (D4⇒ CO1) = 90.15% and Confidence (D3⇒ CO1) = 72.94%, it can be found that compared to δs, the change in density has a greater impact on δzs. The confidence of (H1 ∩ P3⇒ CO2) = 70.23% reveals that if the burial depth of high plasticity loess soils is below 3 m, the probability of slight collapsibility is only 4.42%. Table 4 lists the association rules for mining category (2). The support range for each rule in the list is 2.60–0.86%, and the confidence is 100%. It can be seen that for the association rule to be 100% confidence, at least two constraints are required. This means that the accuracy of predicting collapsibility by a single factor is not enough. Among these, (1): For δs, the Support (E1 ∩ G4⇒ C1) = 2.60% and Confidence (E1 ∩ G4⇒ C1) = 100% indicates that there are 2.60% of cases with the geostatic stress greater than 252.3 kPa with void ratio ≤ 0.8106, all of which exhibit non-collapsibility. The Confidence (D3 ∩ E1 ∩ G4⇒ C1) = 100% also indicates that if the natural density = D3, void ratio = E1 and geostatic stress = G2, there are non-collapsibility. The Support (D2 ∩ E3 ∩ G3 ∩ S3⇒ C2) = 100% indicates that if the medium density and high void ratio soil has a saturation of 33.5–48%, when the geostatic stress is at 164.3–252.3 kPa, it will easily occur slight collapsibility. (2): For δzs, the Confidence (H4 ∩ W4⇒ CO1) = 100% indicates that if the water content is greater than 20.4% and the burial depth exceeds 9 m, it is most likely to be non-collapsible. According to Table 4, these association rules with 100% confidence can contribute to us determining collapsibility. For δs (coefficient of collapsibility), it can be determined as non-collapsibility when any of the following conditions occur: ① Void ratio = E1(≤ 0.816) and Geostatic stress = G4(> 252.3kpa), ② Burial depth = H4(> 9 m) and Saturation = S4(> 67.8%), ③ Plasticity index = P1(≤ 7.3) and Saturation = S4(> 67.8%), ④ Natural density = D4(> 1.8 g/cm^3^) and Plasticity index = P1(< 7.3). In addition, when the Saturation is 33.5–48% and the Geostatic stress is 164.3–252.3kpa, the loess sample is 83% likely to be slight-collapsibility. For δzs (coefficient of collapsibility under overburden pressure), it can be determined as non-collapsibility when any of the following conditions occur: ① Void ratio = E01(≤ 0.789) and Plasticity index = P1(≤ 7.3), ② Burial depth = H4(> 9 m) and Water content = W4(> 20.4%). Conversely, there is a 70% possibility that the loess is slight-collapsibility when the burial depth is less than 3 m and the plasticity index is 8.2–10.2. If the natural density of the loess is greater than 1.8 g/cm^3^ and the plasticity index is less than 7.3, then both δs and δzs are non-collapsibility. However, when the natural density is 1.54–1.66 g/cm^3^ and geostatic stress is 164.3–252.3kpa, the possibility of slight-collapsibility of δs and δzs is 86%.

**Table 4:** Association rules between influence factors and collapsibility (high confidence with relatively high support).

No.	If (influence factors)	Then (δs)	Support	Confidence
1	Void ratio = E1(≤ 0.816) and Geostatic stress = G4(> 252.3kpa)	δs = C1 (non)	2.60	100
2	Density = D3(1.66–1.8 g/cm³) and Void ratio = E1(≤ 0.816) and Geostatic stress = G4(> 252.3kpa)	δs = C1 (non)	2.02	100
3	Void ratio = E1(≤ 0.816) and Geostatic stress = G4(> 252.3kpa) and Saturation = S3(48–67.6%)	δs = C1 (non)	1.64	100
4	Density = D3(1.66–1.8 g/cm³) and Void ratio = E1(≤ 0.816) and Geostatic stress = G4(> 252.3kpa) and Saturation = S3(48–67.6%)	δs = C1 (non)	1.44	100
5	Burial Depth = H4(> 9 m) and Saturation = S4(> 67.8%)	δs = C1 (non)	1.25	100
6	Density = D3(1.66–1.8 g/cm³) and Geostatic stress = G4(> 252.3kpa) and Plasticity index = P1(≤ 7.3)	δs = C1 (non)	1.15	100
7	Density = D2(1.54–1.66 g/cm³) and Void ratio = E3(0.929–1.059) and Geostatic stress = G3(164.3–252.3kpa) and Saturation = S2(33.5–48%)	δs = C2 (slight)	1.11	100
8	Void ratio = E1(≤ 0.816) and Geostatic stress = G4(> 252.3kpa) and Plasticity index = P2(7.3–8.2)	δs = C1 (non)	1.06	100
9	Void ratio = E1(≤ 0.816) and Plasticity index = P1(< 7.3) and Saturation = S3(48–67.6%)	δs = C1 (non)	1.01	100
10	Density = D4(> 1.8 g/cm³) and Geostatic stress = G3(164.3–252.3kpa)	δs = C1 (non)	0.96	100
11	Plasticity index = P1(≤ 7.3) and Saturation = S4(> 67.8%)	δs = C1 (non)	0.96	100
12	Density = D4(> 1.8 g/cm³) and Plasticity index = P1(< 7.3)	δs = C1 (non)	0.87	100

No.	If (influence factors)	Then (δzs)	Support	Confidence
1	Void ratio = E01(≤ 0.789) and Plasticity index = P1(≤ 7.3)	δzs = CO1 (non)	1.83	100
2	Void ratio = E01(≤ 0.789) and Geostatic stress = G4(> 252.3kpa)	δzs = CO1 (non)	1.59	100
3	Density = D3(1.66–1.8 g/cm³) and Geostatic stress = G4(> 252.3kpa) and Water content = W2(11–15.3%)	δzs = CO1 (non)	1.49	100
4	Void ratio = E03(≤ 0.789) and Geostatic stress = G4(> 252.3kpa) and Water content = W2(11–15.3%)	δzs = CO1 (non)	1.30	100
5	Burial Depth = H4(> 9 m) and Water content = W4(> 20.4%)	δzs = CO1 (non)	1.25	100
6	Density = D3(1.66–1.8 g/cm³) and Void ratio = E01(≤ 0.789) and Burial Depth = H4(> 9 m)	δzs = CO1 (non)	1.16	100
7	Density = D3(1.66–1.8 g/cm³) and Geostatic stress = G4(> 252.3kpa) and Plasticity index = P1(< 7.3)	δzs = CO1 (non)	1.16	100
8	Burial Depth = H4(> 9 m) and Density = D4(> 1.8 g/cm³) and Water content = W4(> 20.4%)	δzs = CO1 (non)	1.06	100
9	Water content = W4(> 20.4%) and Geostatic stress = G3(164.3–252.3kpa)	δzs = CO1 (non)	1.01	100
10	Void ratio = E01(≤ 0.789) and Plasticity index = P4(> 10.2)	δzs = CO1 (non)	0.91	100
11	Density = D4(> 1.8 g/cm³) and Plasticity index = P1(≤ 7.3)	δzs = CO1 (non)	0.87	100
12	Density = D3(1.66–1.8 g/cm³) and Void ratio = E01(≤ 0.789) and Plasticity index = P1(≤ 7.3)	δzs = CO1 (non)	0.86	100

**Figure 6:**
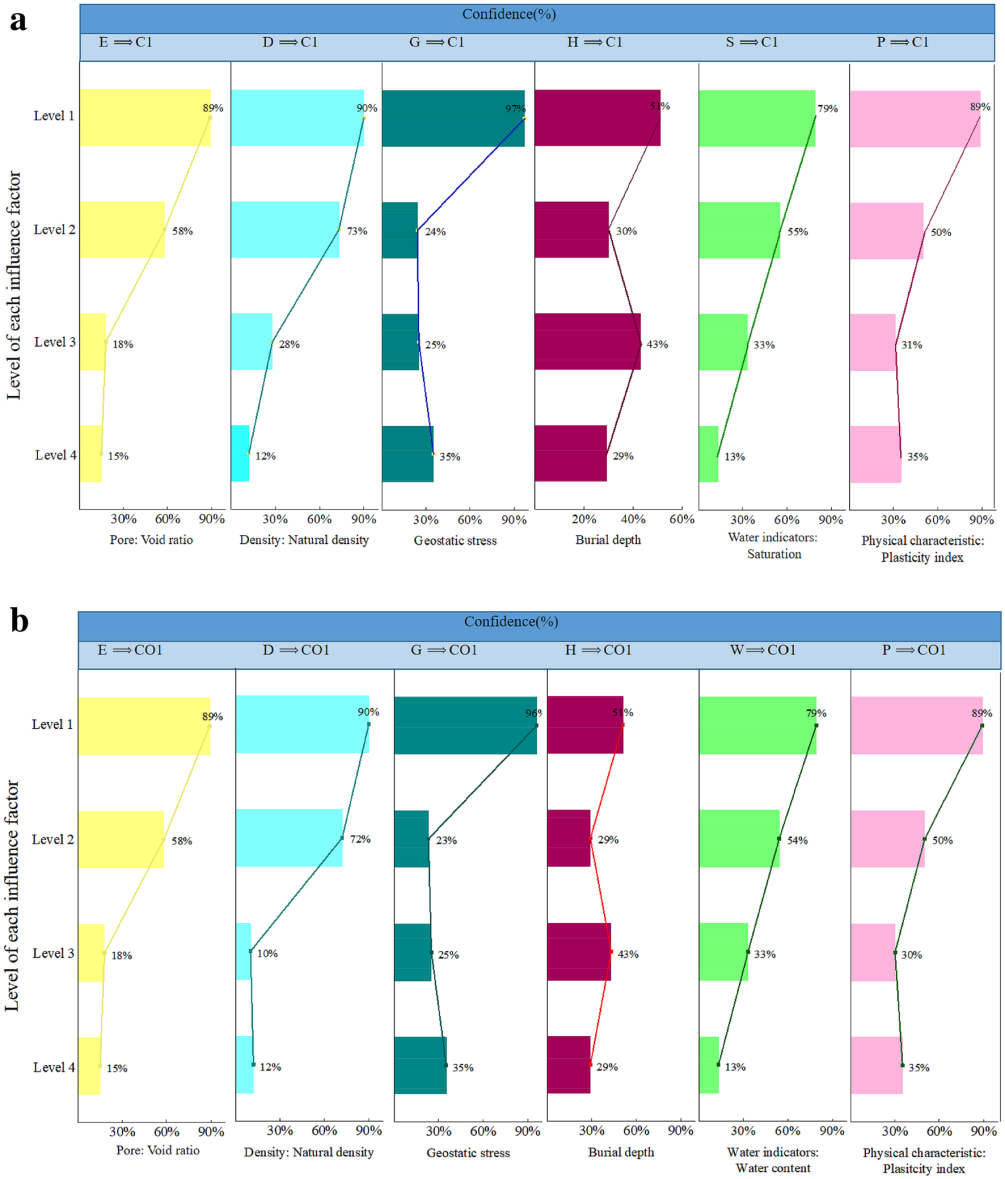
(**a**) Association rules between individual factors and δs within each category. (**b**) Association rules between individual factors and δzs within each category.

For the convenience of single factor analysis, Fig. 6 summarizes the confidence values that each factor is in the first to fourth levels when the loess is non-collapsibility (C1 and CO1).

It can be summarized by analyzing the second column of Fig. 6b that for δzs, with the factor D degraded from level 4(most unfavorable conditions) to level 1(most favorable conditions), the confidence of condition CO1 increases from 12 to 90%. This means that as the natural density increases, the risk of collapsibility correspondingly decreases. A similar trend is also manifested in factor E. If the void ratio of the loess soils is lower than 0.789, the probability of non-collapsibility is calculated to be 88%. In contrast, if the void ratio is more than 1.012, the probability of non-collapsibility declines to 15%. Compared with the condition of H2 and H4, there have higher confidence values when the factor H is in the H1 and H3, which can be attributed that the soils buried at depths 6–9 m or less than 3 m have more serious collapsibility. There is no discernible differentiation between confidence (P1⇒ CO1), confidence (P1⇒ CO1) and confidence (P3⇒ CO1), but the confidence (P4⇒ CO1) is much higher. A reasonable explanation for this result is that when the plasticity index is ≤ 10.2, the possibility of collapsibility will significantly increase. For δs (Fig. 6a), it can be seen that there is the same development tendency of confidence for factors D, G, P and H.

**Figure 7:**
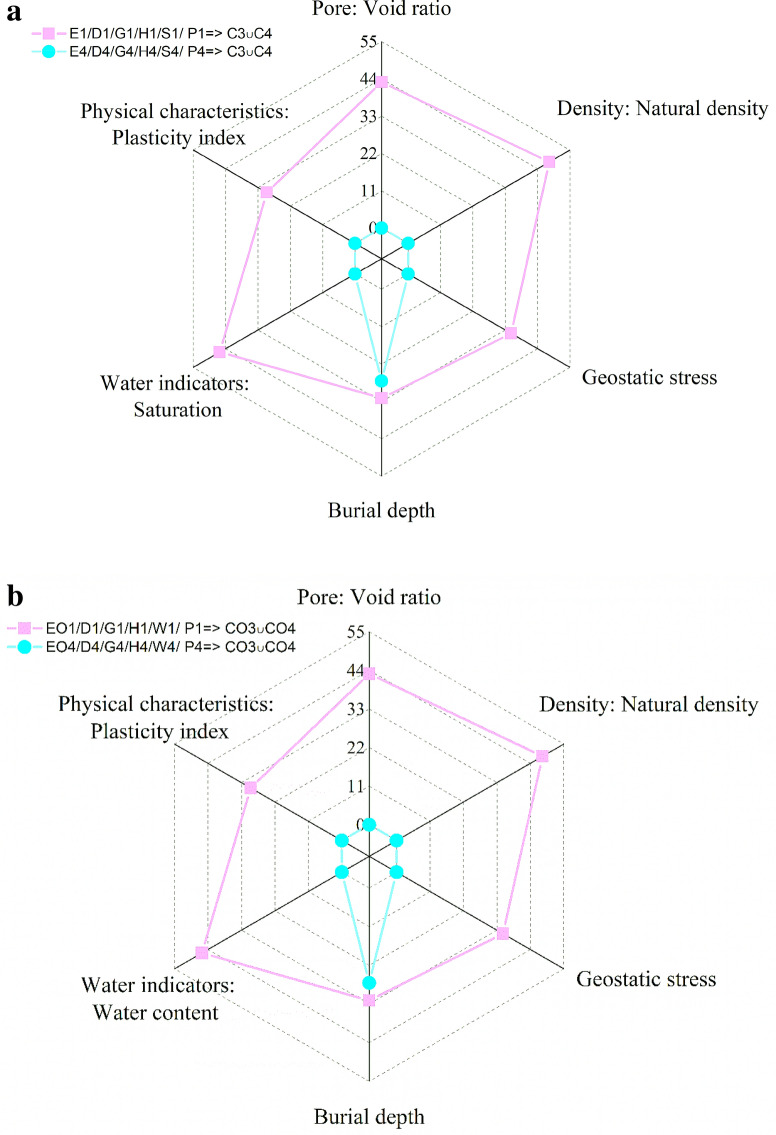
(**a**) Significant extracted association rules when δs = C3 or C4. (**b**) Significant extracted association rules when δzs = CO3 or CO4.

When the collapsibility is in the serious condition (C3, C4 or CO3, CO4), the confidence of each factor under the most favorable conditions and the most unfavorable conditions is shown in Fig. 7. As can be seen from the Fig. 7a, E1/D1/G1/H1/S1/ P1⇒ C3 ∪ C4 is significantly smaller than E4/D4/G4/H4/S4/ P4⇒ C3 ∪ C4, which means that when the physical parameters of the loess reach a certain threshold, the risk caused by δs will increase to a large proportion. On the contrary, it can be concluded that when the physical parameters are below a certain threshold, it is little or no serious risk of collapsing. As Fig. 7a,b shows, we can conclude that, among all the factors studied, natural density is the key factor leading to serious collapsibility. If the natural density increases from under 1.059 g/cm^3^ to above 1.8 g/cm^3^, the probability of C3 or C4 will decrease from 48 to 0%. Instead, burial depth has little effect on collapsibility, with a probability level from 32 to 27%. It can be seen from Fig. 7b, for δzs, there are similar results. But it is worth noting that the probability of CO3 or CO4 in the worst case is less than δs as a whole. In addition, the influence of burial depth on δzs is opposite to that of δs.

## Conclusion

In this paper, the data sets used for the study included 13 influencing factors and 1039 samples from six construction projects in Chengbei District, Xining City, Qinghai Province, China. Then, Apriori algorithm is used to find multiple association rules of collapsibility of loess. The following conclusions can be drawn:

- The potential factors can be divided into six categories according to characteristics, including water indicators, density, pore, burial depth, geostatic stress, and physical characteristics to analyze the influence of these factors on the collapsibility of loess. The original data contains 13 potential influencing factors from six engineering construction projects in Chengbei District, Xining City, Qinghai Province, where the collapsibility of the loess has a great negative impact on engineering design and construction.
- Analyze the key influence factors on δs(coefficient of collapsibility) and δzs(coefficient of collapsibility under overburden pressure), and explore the association rules of the collapsible level in this area. These strong association rules can provide assistance for future research on collapsibility.
- According to Grey Relational Analysis, the key influencing factors in each category are identified. Results indicated that the saturation, natural density, void ratio, plasticity index, geostatic stress, burial depth were the key influence factors to δs. For δzs, the key influence factors are the water content, natural density, porosity, plasticity index, geostatic stress, burial depth. Subsequently, take key factors, δs and δzs as input item, and the Apriori algorithm is used to find multiple association rules. At the same time, the determination of key factors also provides suggestions for the study of predicting δs and δzs.
- In the construction and design of engineering projects in this area, it should be noted that the loess with a burial depth of 6–9 m and less than 3 m in the study area has higher collapsibility. In addition, it is worth mentioning that natural density is the most critical factor leading to collapsibility among physical parameters.
- By using the Apriori algorithm, some strong correlation rules about collapsibility of loess were found. According to those association rules, the evaluation criteria for collapsibility in this area is proposed, which can be used to simplify the workload of determining collapsibility. For example, the engineers can determine that the loess sample is non-collapsible when the geostatic stress is greater than 253.4 kPa and the void ratio is less than 0.816. If the natural density of the soil sample is 1.54–1.66 g/cm^3^ and geostatic stress is 164.3–252.3kpa, then there is an 86% probability of being slight-collapsibility.
